# Supplementary figures and images for: Improving the production of baculovirus expression vector by overexpression of IE0/IE1 through tandem promoter
Source: PLoS One. 2025 Mar 25;20(3):e0320182. doi: 10.1371/journal.pone.0320182 (PMC11936250; doi:10.1371/journal.pone.0320182)

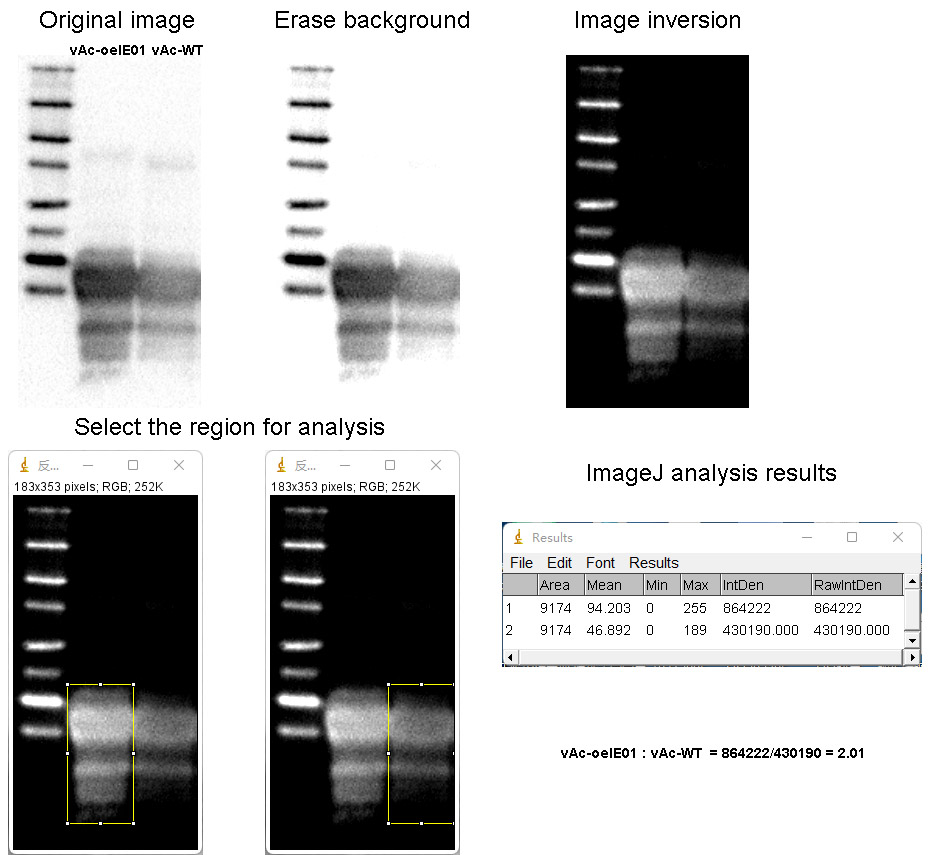

Supplement: S1 Fig — Infect sf9 cells with vAc-WT and vAc-oeIE01 at an MOI of 5 for 5 dpi. GFP was detected through Western blot using a GFP-specific antibody and an HRP-conjugated Goat Anti-Rabbit antibody were used as secondary antibody. Quantification of GFP signals of vAc-WT and vAc-oeIE01 by performing density scans, and ImageJ software was used to analyze and present the results. (JPG) [file pone.0320182.s001.jpg]

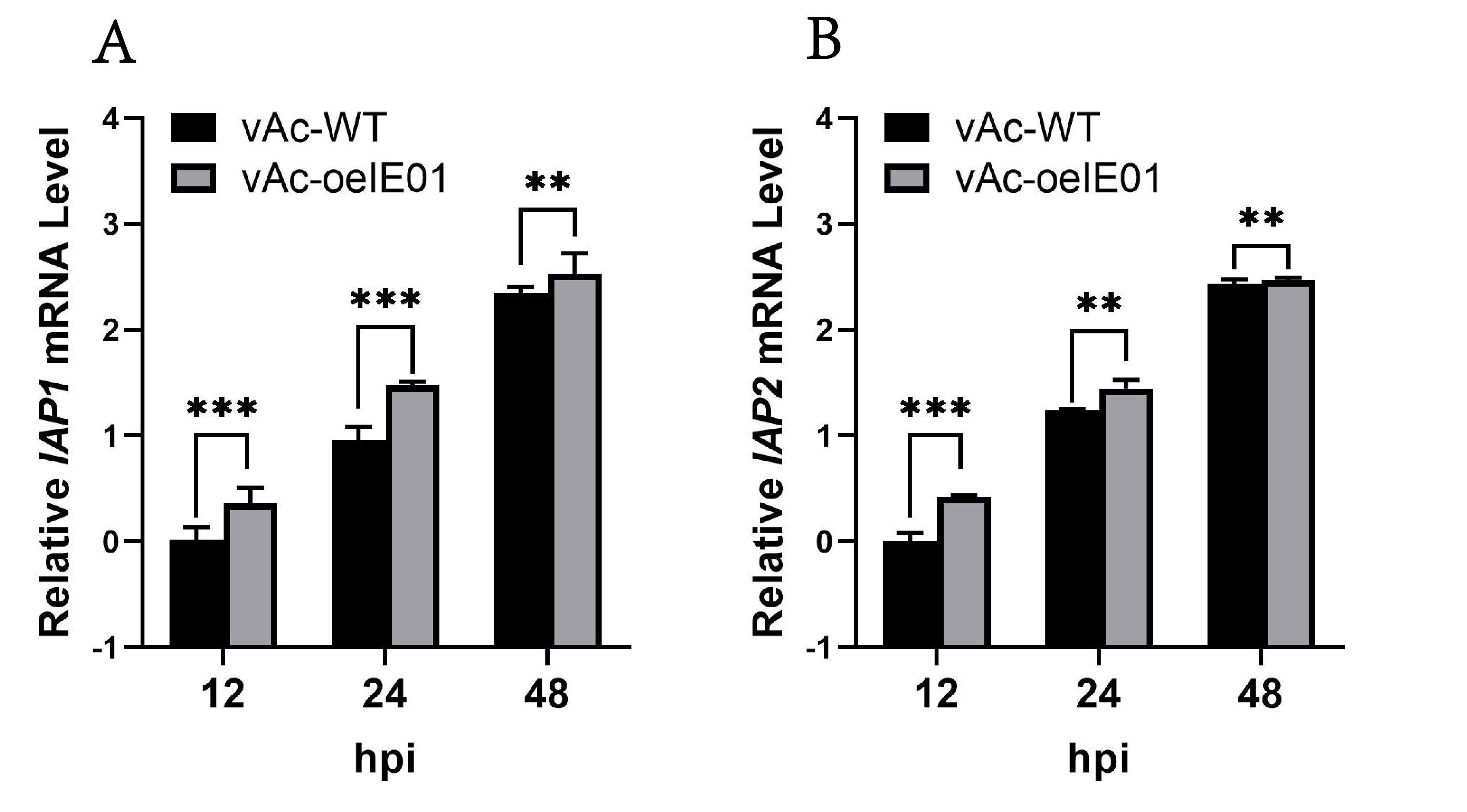

Supplement: S2 Fig — (A) The mRNA level of IAP1 in virus-infected cells were measured at 12-48 hpi. (B) The mRNA level of IAP2 in virus-infected cells were measured at 12-48 hpi. Total RNA samples were extracted from virus-infected cells at 12-48 hpi. The levels of IAP1/IAP2 mRNA were determined with RT-qPCR using ecd mRNA as the internal control. The following primers were used for RT-qPCR: IAP1: IAP1-U (GCAAAGTCTGTCTCGAACGC) and IAP1-D (ACGACACGTCGGACACTTTT); IAP2: IAP2-U (GCCGGCACAAACAAAATTGC) and IAP2-D (AGGAATCAAATCGGCAGCCA). (**p ≤ 0.01; ***p ≤ 0.001). (JPG) [file pone.0320182.s002.jpg]
