# Supplementary material for: Improving the production of baculovirus expression vector by overexpression of IE0/IE1 through tandem promoter
Source: PLoS One. 2025 Mar 25;20(3):e0320182. doi: 10.1371/journal.pone.0320182 (PMC11936250; doi:10.1371/journal.pone.0320182)
Supplement: S1 Raw image — (PDF) [file pone.0320182.s003.pdf]

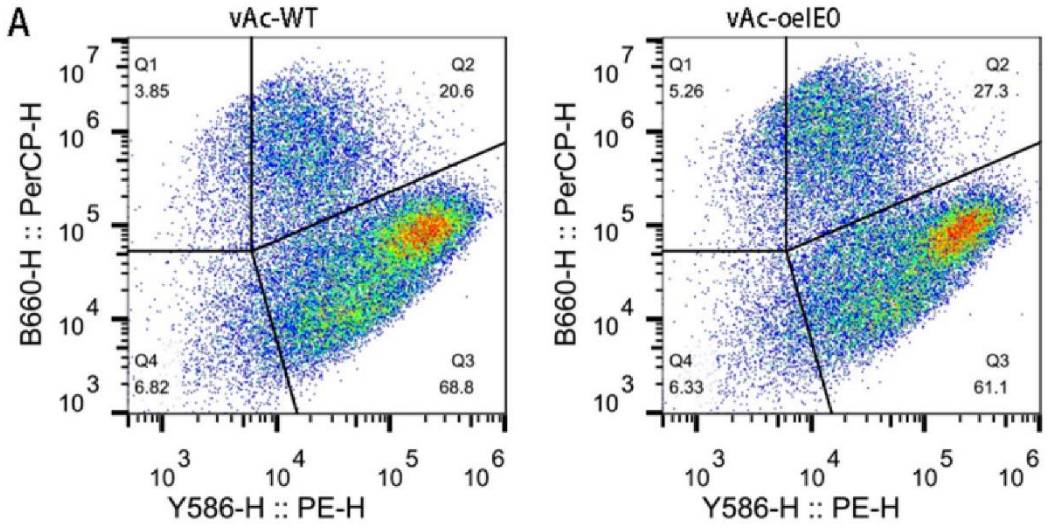

Fig 4A. Apoptosis analysis by flow cytometry. Cells infected with vAc-WT and vAc-oeIE01 were collected at 3 dpi using a flow cytometer （Agilent） and analyzed using FlowJo software. The images were directly exported from FlowJo, with identity information labeled. The images have not undergone any post-processing to ensure the authenticity of the raw data.

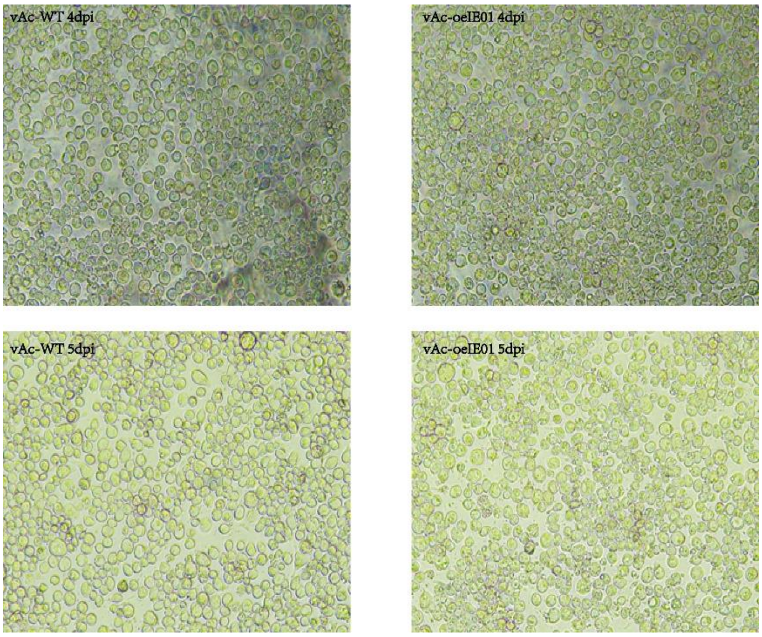

Fig 4B. Cell morphological changes in infected vAc-WT and vAc-oeIE01 at 4-5 dpi. The cell morphology images captured using a Leica optical microscope clearly demonstrate cellular changes. The four images respectively show the morphological changes of *sf9* cells at 4-5 dpi infected with

vAc-WT and vAc-oeIE01. All images have been labeled with their identities and have not undergone any post-processing to ensure the authenticity of the raw data.

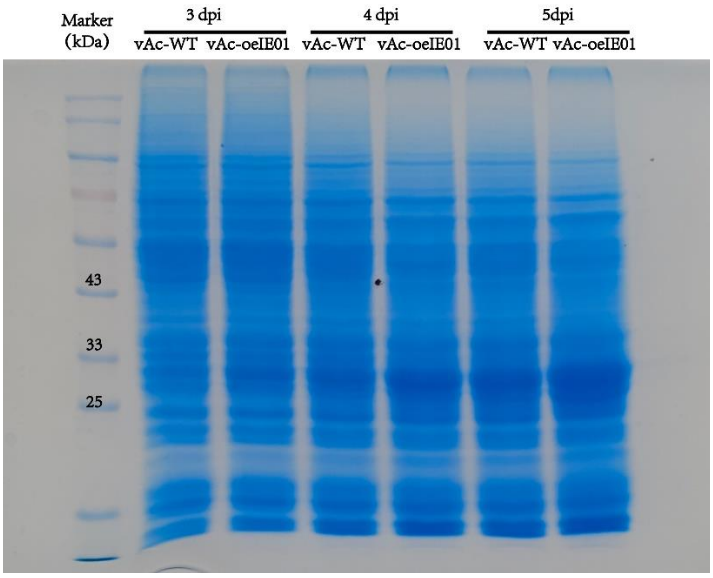

Fig 5A. The relationship between GFP production and viral infection time. The SDS-PAGE analysis image captured using a mobile phone camera is clear and detailed. The image demonstrates the separation of samples: Lane 1 represents the molecular weight marker, and Lanes 2-7 correspond to samples from different treatment groups. The loading order and sample identities for all lanes have been labeled. The image has not undergone any post-processing to ensure the authenticity of the raw data.

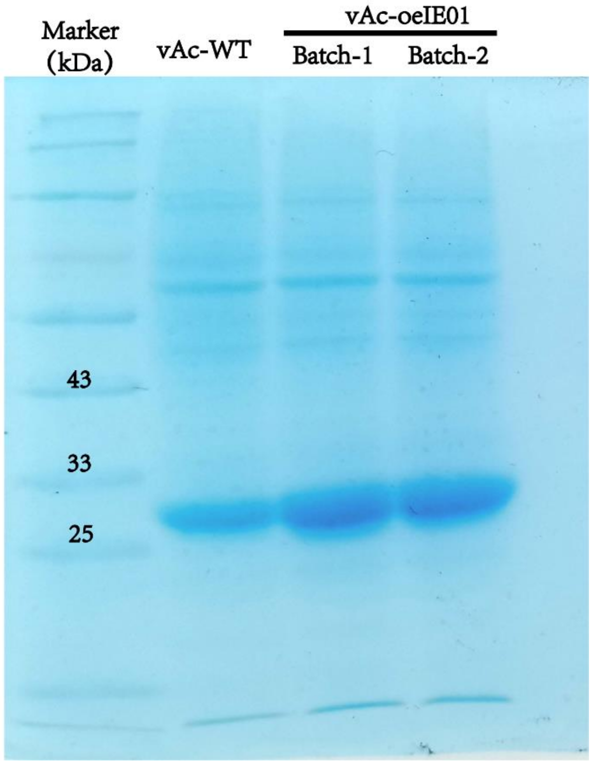

Fig 5C. Detection of the GFP protein levels by SDS-PAGE at 5 dpi. The SDS-PAGE analysis image captured using a mobile phone camera is clear and detailed. The image demonstrates the separation of samples: Lane 1 represents the molecular weight marker, and Lanes 2-4 correspond to samples from different treatment groups. The loading order and sample identities for all lanes have been labeled. The image has not undergone any post-processing to ensure the authenticity of the raw data.

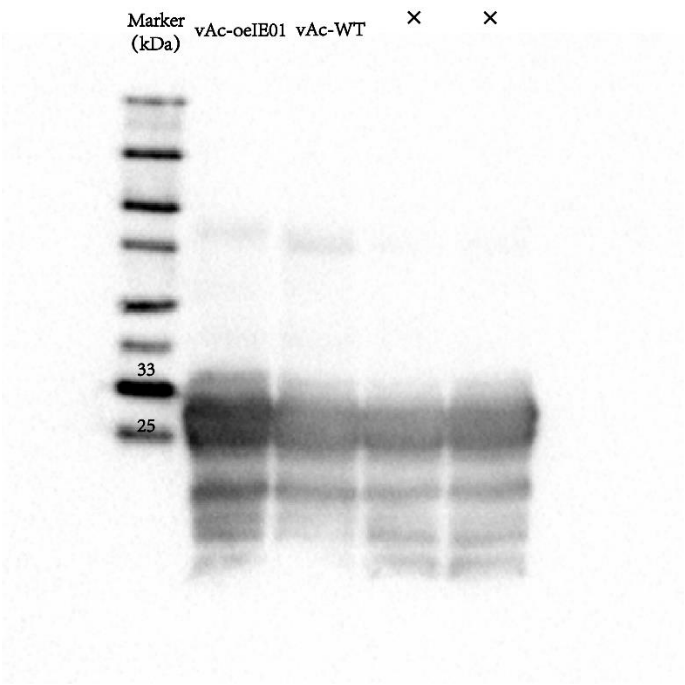

S1 Fig. Western Blot Analysis of GFP Expression in *sf9* cells at 5 dpi. Western blot gel images were captured using a chemiluminescence gel imaging system (Bio-Rad) and analyzed with Image Lab software. The loading order and sample identities for all lanes have been labeled. The images are clear with distinct details and have not undergone any post-processing to ensure the authenticity of the raw data.
