# Supplementary material for: Improving the production of baculovirus expression vector by overexpression of IE0/IE1 through tandem promoter
Source: PLoS One. 2025 Mar 25;20(3):e0320182. doi: 10.1371/journal.pone.0320182 (PMC11936250; doi:10.1371/journal.pone.0320182)
Supplement: Attachment — Submitted filename: Response to Reviewers 2.docx [file pone.0320182.s006.docx]

Xiaodong Xu, PhD

Northwest A&F University

Yangling, Shaanxi, 712100

China

Email: [xuxd@nwsuaf.edu.cn](mailto:xuxd@nwsuaf.edu.cn)

February 13, 2025

Dear Editors and Reviewers:

We sincerely thank the reviewer for the careful review and valuable comments on our study. The suggestion to perform Western blot analysis of IE0, IE1, and IAPs to compare their protein expression levels is highly important. We fully agree that an increase in mRNA levels does not always correlate with an increase in protein abundance, and thus, it is essential to validate protein expression levels.

However, due to the lack of commercially available specific antibodies against IE0, IE1, and IAPs, we are currently unable to directly perform Western blot analysis for these proteins. Nevertheless, to address the reviewer’s suggestion as much as possible, we detected the transcription levels of IAP1 and IAP2 in both experimental and control groups within 12-48 hpi (Fig S2). The results demonstrated that overexpression of IE01 increased the transcription levels of IAP1 and IAP2. As mentioned in our manuscript, IE01, as an earliest pivotal regulatory factor of the baculovirus, exerts a global influence on the expression levels of downstream genes when overexpressed. Consequently, the observed enhance in the expression levels of IAP1 and IAP2 aligns with our expectations. In line with your previous conjecture, the expression capacity of the *vp39* promoter we adopted is significantly lower compared with that of the late promoters *p10* and *polh*. Although the elevated levels of IAPs enhance the anti-apoptotic capacity of cells, this enhancement is insufficient to fully counteract the apoptotic effects triggered by overexpression of IE01.

Meanwhile, we also agree with your opinion that changes in the signal intensity of Western blot bands do not necessarily directly reflect proportional differences in the quantity of proteins. The supplementary Fig.S1 we provided previously was mainly intended to further enrich the data support for the increase in protein expression levels. We will carefully adjust the relevant expressions in the article to ensure the accuracy and rigor of the wording.

We have made appropriate revisions to the other detailed questions you raised about the manuscript. Once again, we sincerely appreciate the insightful comments you provided, which have been of great help during the revision process of our manuscript and significantly improved its quality.

This study was financially supported by two grants:

1. Shaanxi Province Key Research and Development Program (Project No. 2022QCY-LL-52), recipient: Hao Nan
2. A collaborative project with Shaanxi Sky Pet Biotechnology Co., Ltd. (Project No. K4050722064), recipient: Wangcheng Song

**The funders had no role in study design, data collection and analysis, decision to publish, or preparation of the manuscript.**

We have completed the required revisions in the Cover Letter and re-uploaded the documents accordingly. We kindly request your assistance in updating the funding information in the online submission system.

**S2 Fig.** **Insertion of the *vp39* promoter has enhanced the expression of IAPs.** (A) The mRNA level of *IAP1* in virus-infected cells were measured at 12-48 hpi. (B) The mRNA level of *IAP2* in virus-infected cells were measured at 12-48 hpi. Total RNA samples were extracted from virus-infected cells at 12-48 hpi. The levels of *IAP1*/*IAP2* mRNA were determined with RT-qPCR using *ecd* mRNA as the internal control. The following primers were used for RT-qPCR: IAP1: IAP1-U (GCAAAGTCTGTCTCGAACGC) and IAP1-D (ACGACACGTCGGACACTTTT); IAP2: IAP2-U (GCCGGCACAAACAAAATTGC) and IAP2-D (AGGAATCAAATCGGCAGCCA). (**p≤0.01; ***p≤0.001).
